# A Schelling model with adaptive tolerance

**DOI:** 10.1371/journal.pone.0193950

**Published:** 2018-03-06

**Authors:** Linda Urselmans, Steve Phelps

**Affiliations:** 1 University of Essex, Colchester, United Kingdom; 2 King’s College London, London, United Kingdom; Universite Toulouse 1 Capitole, FRANCE

## Abstract

We introduce a Schelling model in which people are modelled as agents following simple behavioural rules which dictate their tolerance to others, their corresponding preference for particular locations, and in turn their movement through a geographic or social space. Our innovation over previous work is to allow agents to *adapt* their tolerance to others in response to their local environment, in line with contemporary theories from social psychology. We show that adaptive tolerance leads to a polarization in tolerance levels, with distinct modes at either extreme of the distribution. Moreover, agents self-organize into communities of like-tolerance, just as they congregate with those of same colour. Our results are robust not only to variations in free parameters, but also experimental treatments in which migrants are dynamically introduced into the native population. We argue that this model provides one possible parsimonious explanation of the political landscape circa 2016.

## 1 Introduction

In this paper we model the adaptation of tolerance in reaction to migration flows into an existing population by drawing on related work from social psychology and political science. International migration is becoming an increasingly defining feature of Western countries, and a key question is what the social implications of large-scale migration and increasing ethnic diversity are. As societies grow more diverse and immigration increases as the world becomes increasingly globalised, concerns about immigration in Europe are high [1].

In social psychology, immigrants and citizens of host societies can be understood as two different groups that lend identity to its members. To native-born people, immigrants thus form an ‘outgroup’. Intergroup threat theory describes the perceptions of threat that people perceive from an outgroup [2]. Perceived threats to society and culture from immigrants are worrying large amounts of voters across Europe [1]. Threat theory includes perceptive threats, which will be the focus in this paper. Thus, whether or not the threat is real is not the primary concern: what matters is that people feel *as if* it were real. Survey respondents frequently overestimate the number of immigrants in their country [3]. Actual numbers of migrants do not predict perceived threat [2, 4], but perceived numbers do [4]. This can help explain why anti-immigration attitudes are often high in areas with low migration: following the Brexit referendum in 2016, [5] have examined the demographics of voters. They find a negative relationship between EU migration and support for leaving the EU: “of the 20 places with the most EU migrants 18 voted to remain. In many of the areas that were among the most receptive to the Leave campaign there were hardly any EU migrants at all.” [5, p.10].

Intergroup threat theory has proven an effective tool in testing what drives such sentiments [6]. Perceived threats to key values in society can explain the anti-immigrant hostility in Europe [7]. Those who believe that traditional values are undermined and that societal cohesion is not ‘what it once was’, are also more likely to be sceptical of immigration [7].

Integrated threat theory has drawn elements from intergroup contact theory, initially proposed by [8] in 1954 as the ‘Contact hypothesis’. Drawing on studies from mixed and segregated neighbourhoods in the US, Allport concluded that under certain conditions, white people with frequent contact with black people experienced decreased racial prejudice. Intergroup contact theory is *not* a proposition of *frictionless* interactions of out-groups resulting in increased trust or social cohesion. Positive contact can potentially lead to these outcomes, but negative contact implies opposite effects [9]. Physical proximity increases the likelihood of contact, but whether that contact is positive (promoting understanding) or negative (invoking a threat perception) is not always clear [9]. In many empirical cases which often times feature migrants as an out-group, the contact conditions are not positive [9]. Migrants that flee poverty and seek work in a first-world country may have a very different collective set of common goals than the host society that was born into what they perceive as the status quo. Differing cultural norms between the host and migrant population can present a social challenge to migration [10], and this would not constitute a positive contact situation. Migrants might not speak the native language, presenting an obvious technical barrier to surpass, strengthening the ‘otherness’ perception of out-groups [10].

In the field of political science, contact theory has enjoyed increased attention since Robert Putnam proposed that, contrary to the consensus at the time, diversity decreases social cohesion in communities [11, 12]. Putnam pointed out that while immigration as a source of diversity has a positive effect on society in the long run, its short-term effects can be largely negative. [10] echoes similar sentiments: in the long run, immigrants contribute to society and integrate, but in the short run, positive effects may be outweighed by social friction generated from the influx of diversity.

Thus, threat theory is the study of precedents of prejudice towards outgroups, and contact theory is the study of the context in which different groups interact.

Both theories have been empirically tested in social psychology and political science, resulting in hundreds of studies (for a meta-study of contact and threat theory, see [13], for a meta-study on social capital and cohesion, see [14]). To date, the relationship between diversity and social capital is unclear. The occurrence and strength of the relationship is dependent on the context it is placed in. [13] find whether data is taken from national or sub-national level, will effect the resolution of information of groups and their behaviour and ultimately, the results of a study. Movement is easier to capture with higher resolution data. Tangential to these findings, [15] notes the stark differences in operationalisation of social capital variables (particularly that of trust) that drive the differences in results between studies.

Empirical studies face difficulties in operationalising variables. Effects on the individual level, such as decreased prejudice as a result of positive contact with an out-group member, must not necessarily persist at the group level. [16] However, much of the research that has spawned as a result of [12]’s finding that diversity invoking threat perceptions has been on macro-level. This means that parts of the underlying theory cannot be captured. Threat theory emphasizes the threat-related antecedents of prejudice such as loss of identity though the presence of an outgroup that challenges the values on which the identity is founded. Contact theory by contrast focuses on the context of the contact [2].

We bridge this micro-macro gap by employing an agent-based model [17]. Agent-based models are particularly suited to exploring theories of social complexity since they are able to capture the properties of heterogeneous populations of individuals, each of who act according to realistic behavioural rules and are located in a geographic and/or social space [18]. Typically, agent-based modelling takes a bottom-up approach in which the model is imbued with micro-level behaviours, which then give rise to macro-level behaviours which can be observed empirically in the output from the model; thus it is easier to bridge the micro-macro gap because all micro-level behaviour is automatically accounted for.

This paper approaches inter-group tolerance from an agent-based perspective in order to understand the implications of migration as an introduction of diversity into an existing population. Previous research has employed agent-based models to introduce differing levels of tolerance [19] and to explore the minority-majority relationships of different groups [20]. In this paper we build on the formalization of the model proposed by [19]. We analyse a model in which we introduce two crucial innovations. Firstly, our model incorporates migration. Secondly, tolerance in our model is adaptive; agents can alter their tolerance levels as they evaluate their surroundings. We use our model to investigate segregation outcomes under environmental conditions where migrants introduce new diversity into the existing population, and both groups have to adapt to the changed social environment. The adaptation of the model proposed by [19] was chosen so that model outcomes of non-adaptive and adaptive agents can be compared more easily.

The remainder of the paper is structured as follows. In the next section we describe our methods and our model. In section 3 we present our results. Finally we conclude in section 4.

## 2 Method

We use agent-based modelling [17] to analyse the relationship between diversity and social cohesion. Our model is based on the framework originally introduced by Schelling [21]. Two differently-coloured populations of agents are situated in a 2-dimensional grid on which they can move around. The agents have a preference for locations which are populated by agents of the own colour, and they move accordingly. Preferences are quantified according to the threshold fraction of similarly-coloured agents in the neighbourhood that is required for an agent to be satisfied with its locale. [21] showed that even a small preference to be near agents of the same colour gives rise to a large amount of segregation.

We use a similar framework, but introduce migration and adaptation of tolerance. We denote one of the colours—green—as representing natives, and the other—blue—as representing migrants. Migration is modelled by allowing the blue population to grow as new migrants arrive at particular times, and at particular locations, around which they cluster. Both groups of agents follow the same behavioural rules, which comprise a movement rule, and a tolerance adaptation rule. The former is similar to earlier Schelling models in which agents move over time relocating to their preferred neighbourhoods. The latter is an innovation of our particular model; when agents are exposed to the out-group their tolerance increases if they are currently satisfied with their environment, but otherwise it decreases. The model thus deviates from the original Schelling model to include additions that have contributed to recent literature in Urban Studies [19].

In the next section we describe our model in precise detail. The model is analysed by simulating it very many times, recording and drawing free parameters randomly as described in section 2.2. We analyse the model under five different immigration treatments, which are described in section 2.3. We record the dependent-variables for each simulation run, as described in section 2.4. In section 3 we present a cross-sectional and time-series analysis of dependent and independent variables under each treatment. Our analysis shows that there are very clear effects, which can be demonstrated by the use of simple descriptive statistics and scatter-plots, and without the need to resort to opaque statistical tests. The source-code used for simulations is freely available under an open-source license [22].

### 2.1 The model

**Fig 1:**
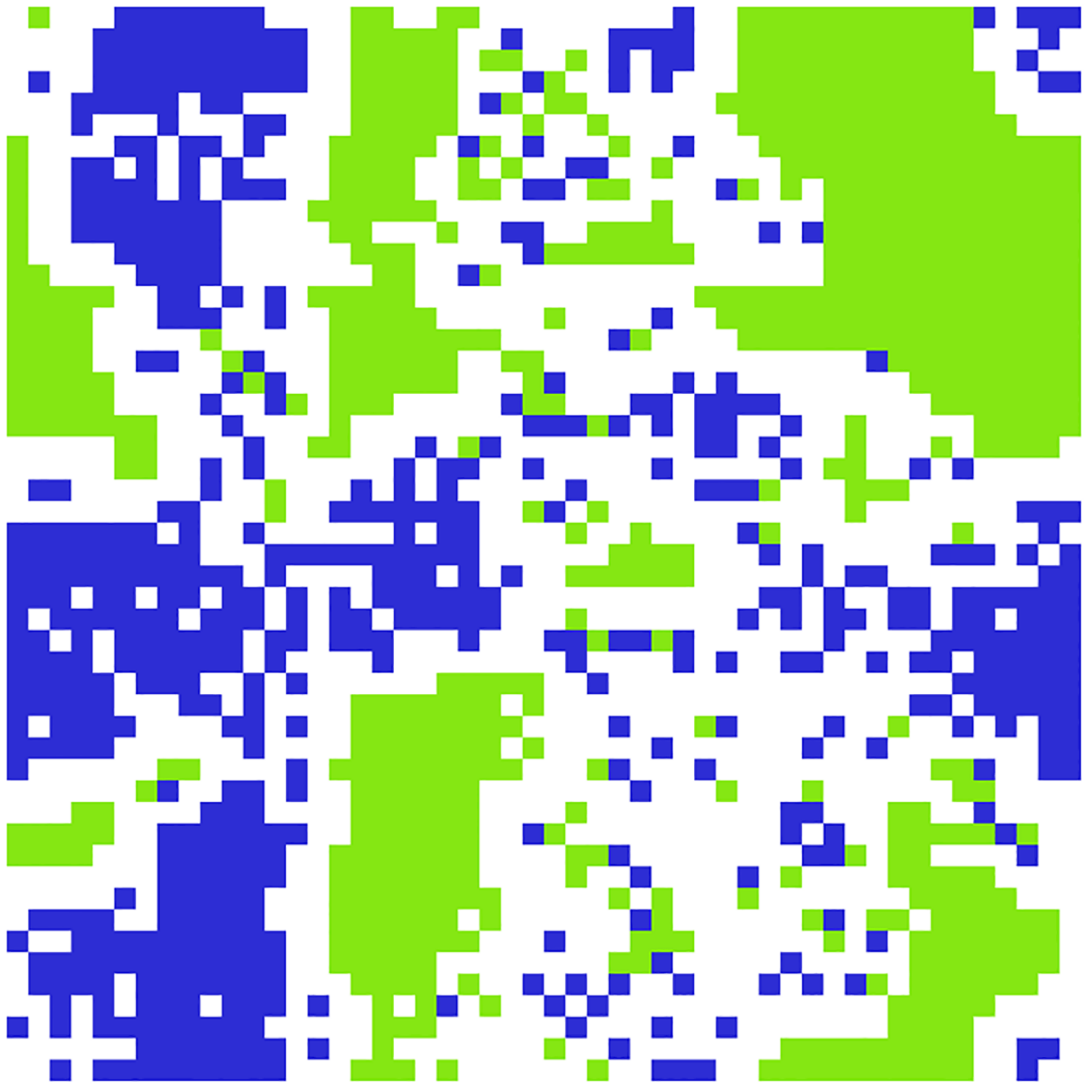
An example state of the simulation showing the colour *c*_*i*_ of each agent *a*_*i*_. Blue squares are occupied by migrant agents and green squares by natives. White squares are empty cells. Both populations eventually form visible clusters.

A set of agents *A*_*t*_ = {*a*_1_, …, *a*_*n*,*t*_} are located on a toroidal lattice with a total of *N* = 50 × 50 vertices *V* at time t∈Z. The location of agent *a*_*i*_ at time *t* is denoted *p*_*i*,*t*_ ∈ *V*. Each agent *a*_*i*_ has a colour attribute denoted *c*_*i*_, which is either blue (*c*_*i*_ = *B*), or green (*c*_*i*_ = *G*). Green agents are the hosts (‘natives’) and they are randomly placed onto the lattice at the beginning of each simulation. Blue agents are migrants and arrive at a later stage. Agents cannot die or otherwise exit the grid. Fig 1 shows a visualisation of a typical state of the model.

Agents have a preference to be near other agents of the same colour, but can see only their local neighbourhood. Formally, let *H*(*p*) denote the 5 × 5 Moore neighbourhood of location *p* ∈ *V*, which consists of the set of all other vertices located on the lattice within a Euclidean distance of two nodes from *p* (allowing for diagonal movement). The neighbours of agent *a*_*i*_ are denoted *N*_*t*_(*a*_*i*_) which contains the set of agents in the neighbourhood of *p*_*i*_—i.e. *N*(*a*_*i*_) = {*a*_*j*_: *p*_*j*_ ∈ *H*(*p*_*i*_)}.

Each agent *a*_*i*_ has a tolerance threshold *f*_*i*,*t*_ ∈ [*f*_*min*_, *f*_*max*_] which determines the fraction of out-group members the agent tolerates in their immediate neighbourhood. The fraction of agents that are similar to agent *a*_*i*_ is given by

$$s_{i,t}=\frac{\left|\{a_{j}\in N_{t}\left(a_{i}\right):c_{i}=c_{j})\}\right|}{|N_{t}\left(a_{i}\right)|}.$$ (1)

Agents are either satisfied or dissatisfied with their neighbours. They are satisfied if and only if the fraction of nearby similar agents meets their tolerance threshold. The utility of agent *i* at time *t* is denoted *u*_*i*,*t*_ ∈ {0, 1} and is given by:

$$\begin{array}{c} u_{i,t}=\{\begin{array}{ccc} 1 & : & s_{i,t}\geq f_{i,t}\\ 0 & : & s_{i,t}<f_{i,t} \end{array}. \end{array}$$ (2)

**Algorithm 1** Movement rule for agent *a*_*i*_

*L* ← RandomlyChooseVacantSites(*z*)  ⊳ choose |*L*| = *z* candidate locations

*L*⋆ ← {*p*_*i*,*t*_}              ⊳ initialise candidate locations

**for all** *l* ∈ **do**

 *g* ← |{*a*_*j*_ ∈ *N*_*t*_(*l*):*c*_*i*_ = *c*_*j*_}|        ⊳ number in-group agents in neighborhood

 *d* ← |{*a*_*j*_ ∈ *N*_*t*_(*l*):*c*_*i*_ ≠ *c*_*j*_}|  ⊳ number of out-group agents in neighborhood

 *s* ← *g*/(*g* + *d*)

 **if** *d* > 0 ∧ *s* ≥ *f*_*i*_ **then**

  *L*⋆ ← *L*⋆ ∪ {*l*}            ⊳ update candidate locations

 **end if**

**end for**

*l*⋆ ← ChooseOneAtRandom(L*)

*p*_*i*,*t*+1_ = *l*⋆                     ⊳ update location

The set of dissatisfied agents is given by *D*_*t*_ = {*a*_*i*_ ∈ *A*_*t*_: *u*_*i*,*t*_ = 0}. At every time period each dissatisfied agent *a*_*i*_ ∈ *D*_*t*_, who is currently located at *p*_*i*,*t*_, randomly samples a number, *z*, of unoccupied locations *L*_*i*_ from the lattice. They then randomly choose a new location from the subset of these for which the ratio of in-group to out-group agents meets their tolerance threshold, i.e. {*l* ∈ *L*_*i*_ ∪ *p*_*i*,*t*_: *s*_*i*,*t*_ ≥ *f*_*i*,*t*_}. If no satisfactory alternative locations are found, then the agent remains at its current location *p*_*i*,*t*_. The movement rule for an agent *a*_*i*_ is summarised by the pseudo-code in Algorithm 1.

As in [23], with probability 10^−2^ per tick, agents that are satisfied will also relocate, this time randomly picking a location from *z* randomly-chosen vacant locations, without considering their utility. This models the fact that people in the real world will move due to a variety of reasons, and not just due to diversity tolerances. If an agent fails to find a new location, the agent’s tolerance will remain unchanged. If the agent is still unhappy in the next time period, it will try and find a new location again.

**Algorithm 2** Decision rule of agent *a*_*i*_

**if** |{*a*_*j*_ ∈ *N*_*t*_(*a*_*i*_): *c*_*j*_ ≠ *c*_*i*_}| > 0 **then** ⊳ at least one outgroup agent in neighbourhood?

 **if** *u*_*i*,*t*_ = 0 **then**                 ⊳ agent is dissatisfied?

  Move agent                   ⊳ see Algorithm 1

  *f*_*i*,*t*+1_ ← *f*_*i*,*t*_               ⊳ tolerance remains the same

 **else**

  *f*_*i*,*t*+1_ ← min(*f*_*i*,*t*_ + Δ_*f*_,*f*_*max*_)           ⊳ increase tolerance by Δ_*f*_

  *p* ← draw randomly from *U*(0,1)

  **if** p ≤ 0.01 **then**        ⊳ satisfied agents move with probability 0.01

   *L* ← RandomlyChooseVacantSites(*z*)

   *p*_*i*,*t*+1_ ← ChooseOneAtRandom(*L*)

  **end if**

 **end if**

**else**

 *f*_*i*,*t*+1_ ← max(*f*_*i*,*t*_ − Δ_*f*_,*f*_*min*_)            ⊳ decrease tolerance by Δ_*f*_

**end if**

A key feature of our model is that the tolerance of an agent adapts to its local environment, and positive contact with out-group agents leads to an increase in tolerance. Accordingly, at each time period every satisfied agent *a*_*i*_ ∈ *A*_*t*_: *u*_*i*,*y*_ = 1 who is exposed to at least one out-group agent in its environment increases its tolerance threshold by a constant term Δ_*f*_, up to the maximum value *f*_*max*_. Tolerance decreases by the same amount if an agent is surrounded by agents of the same colour. In all other cases, the tolerance remains the same:

$$\begin{array}{c} f_{i,t+1}=\{\begin{array}{ccc} \text{min}(f_{i,t}+\Delta_{f},f_{max}) & : & u_{i,t}=1\wedge \left|\left\{a_{j}\in N_{t}\left(a_{i}\right):c_{j}\neq c_{i}\right\}\right|>0\\ \text{max}(f_{i,t}-\Delta_{f},f_{min}) & : & |\{a_{j}\in N_{t}\left(a_{i}\right):c_{j}\neq c_{i}\}|=0\\ f_{i,t} & : & u_{i,t}=0\wedge \left|\left\{a_{j}\in N_{t}\left(a_{i}\right):c_{j}\neq c_{i}\right\}\right|>0 \end{array}. \end{array}$$ (3)

The changes in tolerance are thus tightly linked to states of happiness, and contact is not frictionless. The inclusion of the happiness-condition emulates the fact that integration is potentially a costly process. The costs themselves are not modelled, but rather, the happiness condition acts as a proxy for willingness to pay these costs. A happy person is more willing to engage than an unhappy person. Happiness, we recall, is purely a representation of whether the neighbourhood is satisfactory. The entire decision rule of the agents is summarised by the pseudo-code in Algorithm 2.

Depending on the experimental treatment (see section 2.3 below), the population of agents can grow as new migrants arrive. The number of native agents is always constant, but new migrants can arrive in discrete waves of migration. Migration will only occur during the first *t*_*mig*_ = 1000 ticks. The population dynamics are specified in terms of:

1. the final population density—*PopDen*—which is a parameter that specifies the fraction of occupied sites after all migration event have occurred (at *t* = *t*_*mig*_);
2. the native share of the population—*NatShare*—which specifies the ratio of natives to migrants at the end of the simulation; and
3. the number of waves of migration—*E*—which specifies how many migration events occur.

At the beginning of the simulation a total of *N*_*G*_ native agents are placed randomly onto the lattice, where

$$N_{max}=\text{round}(PopDen\times N)$$ (4)

$$N_{G}=\text{round}(NatShare\times N_{max}),$$ (5)

and for the treatment where there is no dynamic immigration (*E* = 0) a total of *N*_*B*_ migrant agents are also placed randomly, where

$$N_{B}=\text{round}\left(\left(1-NatShare\right)\times N_{max}\right)$$ (6)

On the other hand, in treatments where migration is dynamic (*E* > 0), there are no migrant agents on the lattice at the beginning of the simulation. Rather, the first wave of migration occurs at time 0.05 × *t*_*mig*_, and the subsequent migration waves occur at evenly spaced intervals of 0.9 × *t*_*mig*_/*E* ticks. During each wave of migration an additional number

$$\Delta_{B}=\text{round}\left(N_{B}/E\right)$$ (7)

of migrant agents are simultaneously placed onto the lattice, clustering around a focal vacant location *p*_*B*_ with the highest number of migrants in its neighbourhood, breaking ties randomly:

$$p_{B}=\text{ChooseOneAtRandom}(\underset{v\in V:|\{a_{i}\in A_{t}:p_{i}=v\}|=0}{arg max}|\left\{a_{i}\in N\left(v\right):c_{i}=B\right\}\left|\right)$$ (8)

If there are no existing migrants on the grid, then instead we use the total number of agents in each neighbourhood to rank candidate focal locations. The additional migration sites for the new arrivals are chosen by iteratively finding the best neighbour of the chosen focal location *p*_*B*_; sites are ranked firstly according to the highest number of surrounding new migrants, and secondly according to their local population density within their neighbourhood. The placement algorithm is summarised in the pseudo-code given by algorithms 3 and 4. A visualisation of this process can be found at [24].

**Algorithm 3** Choose locations for migrant agents during migration waves

**function** PlaceMigrants(*p*_*B*_, Δ_*B*_)   ⊳ Place Δ_*B*_ migrant agents around location *p*_*B*_

 *P*_*B*_ ← {*p*_*B*_}            ⊳ Initialise the set of locations for immigration

 **while** |*P*_*B*_| < Δ_*B*_ **do**              ⊳ More migrants to place?

  *p*_*B*_ ← BestNeighbour(*p*_*B*_,*P*_*B*_)      ⊳ Find the best neighbouring location

  *P*_*B*_ ← *P*_*B*_ ∪ *p*_*B*_                ⊳ add it to the result set

 **end** **while**

 **return** *P*_*B*_

**end** **function**

**Algorithm 4** Find the neighbouring site with the greatest population density

**function** BestNeighbour(*p*_*B*_, *P*_*B*_)   ⊳ Best neighbour of *p*_*B*_ excluding locations *P*_*B*_

 **if** |*N*(*p*_*B*_) − *P*_*B*_ − {*a*_*i*_: *p*_*i*_ ∈ *N*(*p*_*B*_)}| > 0 **then**  ⊳ Vacant sites not already chosen?

  P* ← {}                   ⊳ Initialise best locations

  d* ← −∞                   ⊳ Initialise best density

  **for** **all** *p* ∈ *H*(*p*_*B*_) − *P*_*B*_ − {*p*_*i*_: *a*_*i*_ ∈ *A*_*t*_} **do**  ⊳ All vacant unchosen neighbours

   *d* ← |{*a*_*i*_: *p*_*i*_ ∈ *H*(*p*)}|/|*H*(*p*)|      ⊳ Calculate local population density

   **if** *d* > d* **then**

    d* ← *d*

    P* ← P* ∪ (*p*,d*)

   **end** **if**

  **end** **for**

  **return** ChooseOneAtRandom({*p*: (*p*,*d*) ∈ P* ∧ *d* = d*})

 **else**

  *p* ← ChooseOneAtRandom(*H*(*p*_*B*_))

  **return** BestNeighbour(*p*,*P*_*B*_)

 **end** **if**

**end** **function**

### 2.2 Initial conditions

The majority of parameters governing the initial conditions of the model are randomly varied between simulation runs in order to test the robustness of the model. We also record these values so that they can be used as independent variables in order to ascertain any effects. These are described in turn below, and summarised in Table 1 (the remaining constant parameters are summarised in Table 2, and the state variables in Table 3).

**Table 1:** Independent variables.

*Parameter*	*Distribution*	*Description*
*NatShare*	∼ *U*(0.02,0.98)	Fraction of natives
*PopDen*	∼ *U*(0.75,0.98)	Final population density
Δ_*f*_	∼ *U*(10^−5^,10^−3^)	Rate of change of tolerance
*z*	∼ *U*(25,125)	No. of considered locations when moving

**Table 2:** Constants.

*Constant*	*Description*
*t*_*mig*_ = 1,000	Time until final migration
*t*_*max*_ = 20,000	Maximum number of ticks per simulation
*N* = 50 × 50	Size of lattice
*f*_*min*_ = 0.05	Minimum tolerance
*f*_*max*_ = 0.95	Maximum tolerance

**Table 3:** State variables.

*Variable*	*Description*
*A*_*t*_	The population of agents
*c*_*i*,*t*_	Colour of agent *a*_*i*_
*f*_*i*,*t*_	Tolerance of agent *a*_*i*_
*u*_*i*,*t*_	Utility of agent *a*_*i*_
*p*_*i*,*t*_	Position of agent *a*_*i*_
*N*_*t*_(*a*_*i*_)	The set of agents that are neighbours of agent *a*_*i*_
*H*(*p*)	The set of locations in the neighbourhood of location *p*

#### Tolerance distribution

When agent *a*_*i*_ arrives at the simulation its initial tolerance *f*_*i*,0_ is drawn *i.i.d*. from a uniform distribution *f*_*i*,0_ ∼ *U*(*f*_*min*_, *f*_*max*_). After the initialization, the agent adapts their tolerance according to Eq 3 as summarised in Algorithm 2. For all simulations in this paper we set *f*_*min*_ = 0.05 and *f*_*max*_ = 0.95. These limits prevent agents from fixing at the extreme values of tolerance; with these constraints agents will always be able to tolerate one out-group member in their neighbourhood (without these constraints, any agent reaching full tolerance or intolerance would never readjust again, since just one out-group member would be above the tolerance threshold).

#### Rate of change of tolerance

The rate of change of tolerance Δ_*f*_ is the increment used when agents adapt their tolerance (see Eq 3). At the beginning of each simulation it is drawn randomly Δ_*f*_ ∼ *U*(10^−5^, 10^−3^) and remains constant throughout the simulation. The low values of Δ_*f*_ reflect the slow rate of change of attitudes of the population.

#### Final population density

The final population density *PopDen* determines the fraction of occupied sites after all waves of immigration have occurred (|*A*_*t*_*mig*__|/*N*). From thereon, the number of agents is constant. At the beginning of each simulation this parameter is randomly drawn from a uniform distribution ∼*U*(0.75, 0.98).

Schelling models are typically assumed to run under conditions of high density [20], which is why the minimum is still $\frac{3}{4}$ of the map covered. Density also acts as a proxy for freedom of choice. Higher density results in less freedom of choosing better areas.

#### Final native share of the population

The final native share of the population *NatShare* determines the ratio of natives to migrants after all waves of immigration have occurred. This parameter is initialised randomly by drawing from a uniform ∼*U*(0.02, 0.98) at the beginning of each simulation. In treatments without migration (*E* = 0) it determines the fraction of natives in the initial population, which thereafter remains fixed. In treatments with migration (*E* > 0), it determines the number of migrants added in each wave (see Eqs 5, 6 and 7), which in turn determines the final fraction of natives in the population.

Considering the extremes of this parameter, when *NatShare* = 0.02, the world would fill up with migrants until migrants constitute 98% of the population, and natives constitute 2%. Whilst national-level migration does not lead to migrants outnumbering natives, the reasoning is that on smaller geographical areas, this majority-minority flipping can indeed occur. Because segregation is mediated by how society is made up, how big minorities are and how they are distributed, the ratio seeks to test in how far, if at all, different minority-majority relationships influence segregation behaviour and tolerance levels. The traditional Schelling model has usually assumed an even split, an assumption that is not theoretically useful in the context of migration and attitudes towards diversity.

#### Considered tiles to move

The parameter *z* specifies the number of vacant locations than each agent considers when moving. At the beginning of each simulation it is initialised randomly by drawing from a discrete uniform distribution *z* ∼ *U*(25,125). The minimum and maximum of this distribution correspond to 1% and 5% respectively of the size of the entire lattice.

### 2.3 Immigration treatments

There are five different experimental treatments for immigration of blue agents into the model, which correspond to five different values of the parameter *E*. These are summarised in Table 4. Immigration waves arrive within the first 1,000 ticks of *t*_*max*_ = 20,000. This allows agents to adjust their behaviour for a prolonged period after the last migration wave has occurred.

**Table 4:** Treatment conditions.

Treatment	*E* = 0	*E* = 1	*E* = 4	*E* = 15	*E* = 100
**Migration**	No	Yes	Yes	Yes	Yes
**Number of waves**	-	1	4	15	100

The first treatment *E* = 0 is a control condition with no immigration taking place. In this condition, both natives and migrants are initialised at the start of the simulation and there is no increase in the size of the migrant group over time. The four remaining treatments all feature immigration at different rates, aiming to simulate one-off large influxes of migrants as well as a “trickle-down” scenario in which few migrants arrive at one time, but do so for a sustained period of time. The precise dynamics are described by Eqs 4 to 7 in the previous section.

### 2.4 Dependent variables

For each realisation of the model we sample and record dependent variables every 10 time steps, to allow for both cross-sectional and time-series analysis of any effects. These variables are described in turn below, and summarised in Table 5.

**Table 5:** Dependent variables.

*Variable*	*Description*
$M_{t}^{c}$	Segregation of colour at time *t* (Eq 9)
$M_{t}^{f}$	Segregation of tolerance at time *t* (Eq 10)
${\bar{f}}_{B_{t}}$	Tolerance of migrants at time *t* (Eq 11)
${\bar{f}}_{G_{t}}$	Tolerance of natives at time *t* (Eq 12)
*β*_*f*_	Bimodality of tolerance at the end of the simulation (Eq 13)
*M*^*c*^	Segregation of colour at the end of the simulation
*M*^*f*^	Segregation of tolerance at the end of the simulation
${\bar{f}}_{G}$	Tolerance of natives at the end of the simulation
${\bar{f}}_{B}$	Tolerance of migrants at the end of the simulation

As proposed in [23], we record Moran’s index of spatial autocorrelation in order to quantify the amount of segregation by colour:

$$M_{t}^{c}=\frac{|A_{t}|}{\sum_{\left(i,j\right)\in A_{t}^{2}}w_{i,j}}\frac{\sum_{\left(i,j\right)\in A_{t}^{2}}w_{i,j}\left(c_{i}-{\bar{c}}_{t}\right)\left(c_{j}-{\bar{c}}_{t}\right)}{\sum_{i\in A_{t}}{\left(c_{i}-{\bar{c}}_{t}\right)}^{2}}$$ (9)

where the mean colour is ${\bar{c}}_{t}=\sum_{i\in A_{t}}c_{i}/\left|A_{t}\right|$, and *w*_*i*,*j*_ = 1 if and only if agents *a*_*i*_ and *a*_*j*_ are immediately adjacent on the lattice (including diagonals), otherwise *w*_*i*,*j*_ = 0.

Crucially we also compute the Moran’s I of *tolerance*, $M_{t}^{f}$, by substituting *f* in place of *c* in Eq 9:

$$M_{t}^{f}=\frac{|A_{t}|}{\sum_{\left(i,j\right)\in A_{t}^{2}}w_{i,j}}\frac{\sum_{\left(i,j\right)\in A_{t}^{2}}w_{i,j}\left(f_{i}-{\bar{f}}_{t}\right)\left(f_{j}-{\bar{f}}_{t}\right)}{\sum_{i\in A_{t}}{\left(f_{i}-{\bar{f}}_{t}\right)}^{2}}$$ (10)

We also record the first four moments of the tolerance distribution across the population (${\bar{f}}_{t}$, $\sigma_{f_{t}}^{2}$, *γ*_*f*_*t*__ and *κ*_*f*_*t*__), and subdivide this into tolerance of migrants:

$${\bar{f}}_{B_{t}}=\sum_{a_{i}\in B_{t}}f_{i}/\left|B_{t}\right|$$ (11)

and tolerance of natives:

$${\bar{f}}_{G_{t}}=\sum_{a_{i}\in G_{t}}f_{i}/\left|G_{t}\right|$$ (12)

where *B*_*t*_ is the migrant population {*a*_*i*_ ∈ *A*_*t*_: *c*_*i*_ = *B*}, and *G*_*t*_ is the native population {*a*_*i*_ ∈ *A*_*t*_: *c*_*i*_ = *G*}.

Finally, to help identify whether the tolerance distribution is bimodal, we record the bimodality coefficient [25] of the tolerance distribution:

$$\beta_{f_{t}}=\frac{\gamma_{f_{t}}^{2}+1}{\kappa_{f_{t}}}$$ (13)

As a notational convention, we refer to the final value of an independent variable at *t* = *t*_*max*_ by omitting the time subscript from all of the above.

## 3 Results

The model was analysed through simulation and empirical methods. For each treatment in Table 4 we executed 7,000 independent realisations of the model, drawing free parameters from the distributions specified in Table 1. Each realisation was run for a total of *t*_*max*_ = 20,000 simulation ticks. This resulted in a total of 5 × 7,000 = 35,000 cross-sectional samples of each of the dependent variables (Table 5). During each simulation we also sample all dependent-variables every 10 ticks, resulting in a total of 35,000 × (20,000/10) = 7 × 10^7^ time-series samples. In the following we first give an overview of the qualitative properties of a single typical simulation run before analysing the aggregate data across simulation runs. The model is updated sequentially, every tick. Agents act in sequential order; that order is shuffled every tick to avoid tactical advantages that might result from a pre-determined sequence.

### 3.1 A typical simulation run

**Fig 2:**
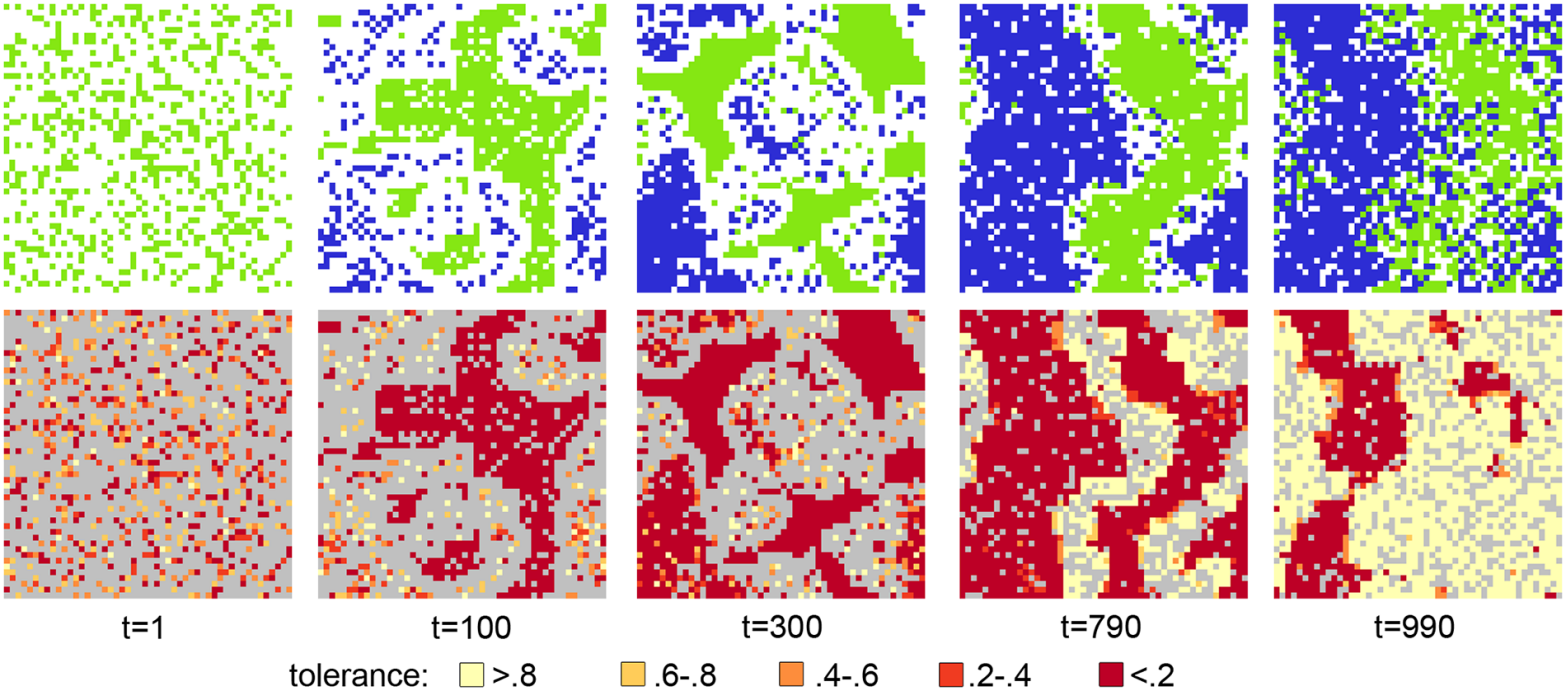
States of the simulation at different times. The top row shows the colours *c*_*i*_ of each agent. The bottom row shows the corresponding tolerance heatmap.

Fig 2 shows a visualisation of a typical simulation run to demonstrate the clustering of agents. The top row shows a progression of migration (*E* = 4) at a starting density of 37%, filling up with migrants up until a 75% density so that both groups are equal in size. The blue and green agents move around, empty space is white.

The bottom row shows the corresponding tolerance heat-map. Light colours denote tolerant agents, dark colours denote intolerant agents, and the grey areas are vacant tiles. The period from *t* = 90 (pre-migration) to *t* = 100 (post-migration) is marked by a significant changes in tolerance levels. The native fraction of the population that is not in vicinity of migrants are uniformally hostile, whereas the newly arrived migrants have a large variance in their tolerance levels, which are randomly drawn from a uniform distribution upon entering the map. At this stage, the map is sparsely populated and the clusters of migrant and natives have visible buffer-zones between which make inter-group contact less likely.

As more migrants arrive, we start to observe the effects of inter-group contact. Natives exposed to migrants react either by increasing their tolerance, resulting in the lighter colours visible at *t* = 300, or by moving. Once all of the migrants arrive, at *t* = 790, there are two pronounced clusters of agents (one of which wraps around the grid). On the tolerance heat-map, we see corresponding clusters of tolerance, with highly-intolerant agents surrounded by highly-tolerant agents. At this stage, most of the population has either very high or very low levels of tolerance, but as long as there is enough empty space to form a buffer zones between the clusters, the majority of agents are still highly intolerant, because only very few contact situations arise; unhappy agents relocate before adapting their tolerance levels.

This situation changes as space becomes more scarce. The final two states shown in Fig 2 illustrate a rapid phase-transition from a mainly intolerant society into a bimodal society of two equally-large fractions of highly-tolerant and highly-intolerant agents. The vacant buffer zones are now populated with tolerant agents of both colours who have relocated from the periphery of their respective clusters, thus forming a new zone of highly-tolerant agents. This tolerant zone expands as more out-group members mix in these high-tolerance areas, which in turn influence agents on the periphery of the intolerant clusters through positive contact, which results in a rapid erosion of their size.

This process continues until the intolerant clusters are completely surrounded by tolerant agents, who are satisfied and therefore static. This provides a dense, rigid substrate which restricts the movement of agents on the periphery of the intolerant clusters, who provide a protective membrane shielding the inner-core from further out-group contact. The periphery itself is highly dynamic; because agents on the periphery are unhappy they relocate, but their range of movement is restricted to locations within or near the cluster. However, inside these clusters, agents are intolerant but satisfied, as they are surrounded by in-group members, and therefore they remain static. Thus the entire cluster of intolerant agents achieves a relatively stable configuration, and persists over time.

The smaller orange-coloured clusters are unstable pockets of medium tolerance which appear throughout the simulation, but rapidly disappear again as they consist entirely of satisfied agents who either become more tolerant through out-group contact, or become isolated and intolerant.

Thus movement and adaptive tolerance interact leading to an emergent shield-and-buffer dynamic that polarises the population, causing it to self-assort along the tolerance axis with agents being either extremely tolerant or intolerant. Although in this section we have only discussed a single simulation run, in subsequent sections we show empirically that the model results in bimodal tolerance for many different initial conditions, and despite Monte-Carlo variance.

### 3.2 Model convergence

As discussed in the previous section, the model exhibits subtle dynamics, and therefore it is important to establish whether the key dependent variables stabilise within the finite time period *t* ≤ *t*_*max*_. We test the convergence of each independent realisation of the model individually by analysing the final *n* = 250 values *V*_*f*_ of the time-series of each independent variable *V* sampled at intervals of 10 ticks. The criteria we use to test convergence of each variable are: i) if the variance of the final sample is extremely small $\sigma_{V_{f}}^{2}<10^{-20}$; or (ii) the standard deviation is small compared to the overall range *σ*_*V*_*f*__ < [max(*V*) − min(*V*)] × 10^−4^; or iii) if *V*_*f*_ is stationary under an augmented Dickey-Fuller test [26]. The latter is established by estimating the model Δ*V*_*t*_ = *α* + *βt* + *γV*_*t*−1_ + *δ*_1_ Δ*V*_*t*−1_ + … + *δ*_λ−1_ Δ*V*_*t*−λ+1_ + *ϵ*_*t*_ where the lag order λ is chosen using the Akaike information criterion, and accepting the time-series as convergent *iff*. if the value of the test statistic $\hat{\gamma }/SE\left(\hat{\gamma }\right)$ is less than the critical value for *p* = 0.05.

These criteria were chosen because they allow us to test not only for cases where the model reaches a static steady state in which values of dependent variables are constant over time, but also stochastic steady states in which the ensemble time-series is stationary; i.e. the *moments*, such as the mean and variance, are constant, despite the fact that the dependent variable has non-zero rate of change.

Using the above criteria, we analyse the time-series of the tolerance of migrants, the tolerance of natives, and the segregation of colour; i.e. $V\in \{{\bar{f}}_{B_{t}},{\bar{f}}_{G_{t}},M_{t}^{c}\}$. We record that the sample path has converged for a given realisation *i*.*i*.*f*. all three variables converge in the final period.

**Fig 3:**
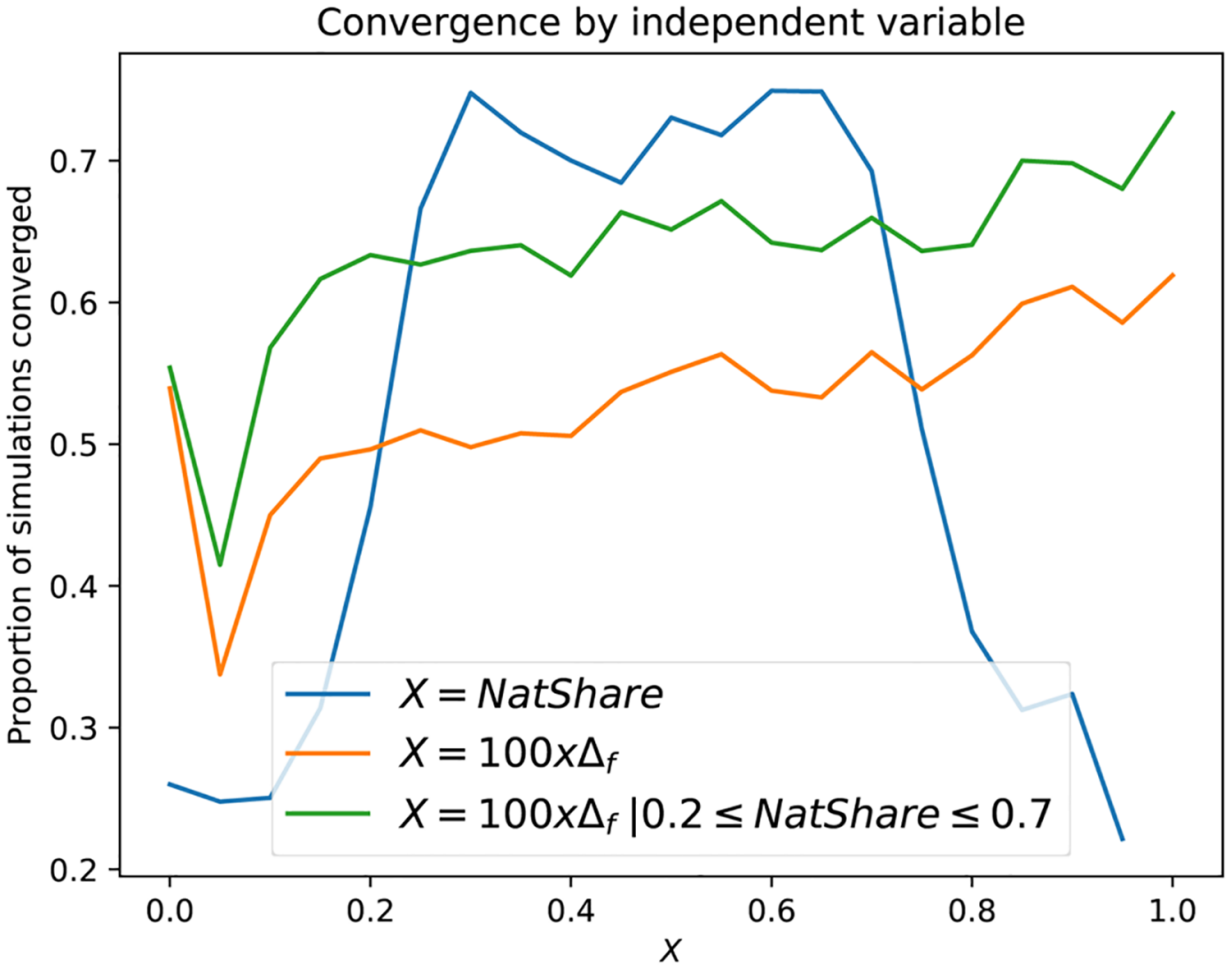
Proportion of sample paths that test positively for convergence by independent variable. Each variable *X* is binned into intervals of size 0.05, and then we count the fraction of sample paths which pass the test within each bin.

Over the entire range of parameters, we were only able to reject the null hypothesis of non-stationarity for 86% of the sample paths. We were able to identify which independent variables contributed to the convergence of the model through a correlation analysis, which identified *NatShare* and Δ_*f*_ as the most promising explanatory variables. We binned *NatShare* and Δ_*f*_ × 100 into bins of size 0.05, and plotted the proportion of sample paths that converged within each bin (Fig 3).

As we expected, the highest failure rate occurs for extreme values of *NatShare* and for small values of Δ_*f*_ < 0.0005; for very small values of Δ_*f*_ agents adapt very slowly, and the model fails to reach equilibrium within *t* ≤ *t*_*max*_, whereas for extreme values of *NatShare*, the respective minorities are likely too small to form sustainable clusters, thus breaking up and forming again. In fact, both variables interact; if we do not control for *NatShare* then the convergence rate increases asymptotically, but slowly, with Δ_*f*_. However, if we control for extreme values of *NatShare* then provided that Δ_*f*_ ≥ 0.0005 we obtain fairly consistent convergence rates of ∼97%. As we show empirically in the next section, for these parameter ranges the ensemble of sample paths is wide-sense stationary; i.e. the mean of the dependent variable across sample paths does not change over time.

Based on our convergence analysis, we restricted the ranges of the *NatShare* and Δ_*f*_ parameters used in the remainder of the paper. All results in subsequent sections use the ranges 0.2 ≤NatShare≤ 0.8, and Δ_*f*_ ≥ 0.0005, for which the vast majority (97%) of sample paths test positively for convergence. These ranges are summarised in Table 6.

**Table 6:** Restricted ranges of independent variables for which the model converges within *t* ≤ *t*_*max*_.

*Parameter*	*Range*
*NatShare*	0.2 ≤ *NatShare* ≤ 0.8
Δ_*f*_	5 × 10^−4^ ≤ Δ_*f*_ < 0.001

### 3.3 Time-series analysis

**Fig 4:**
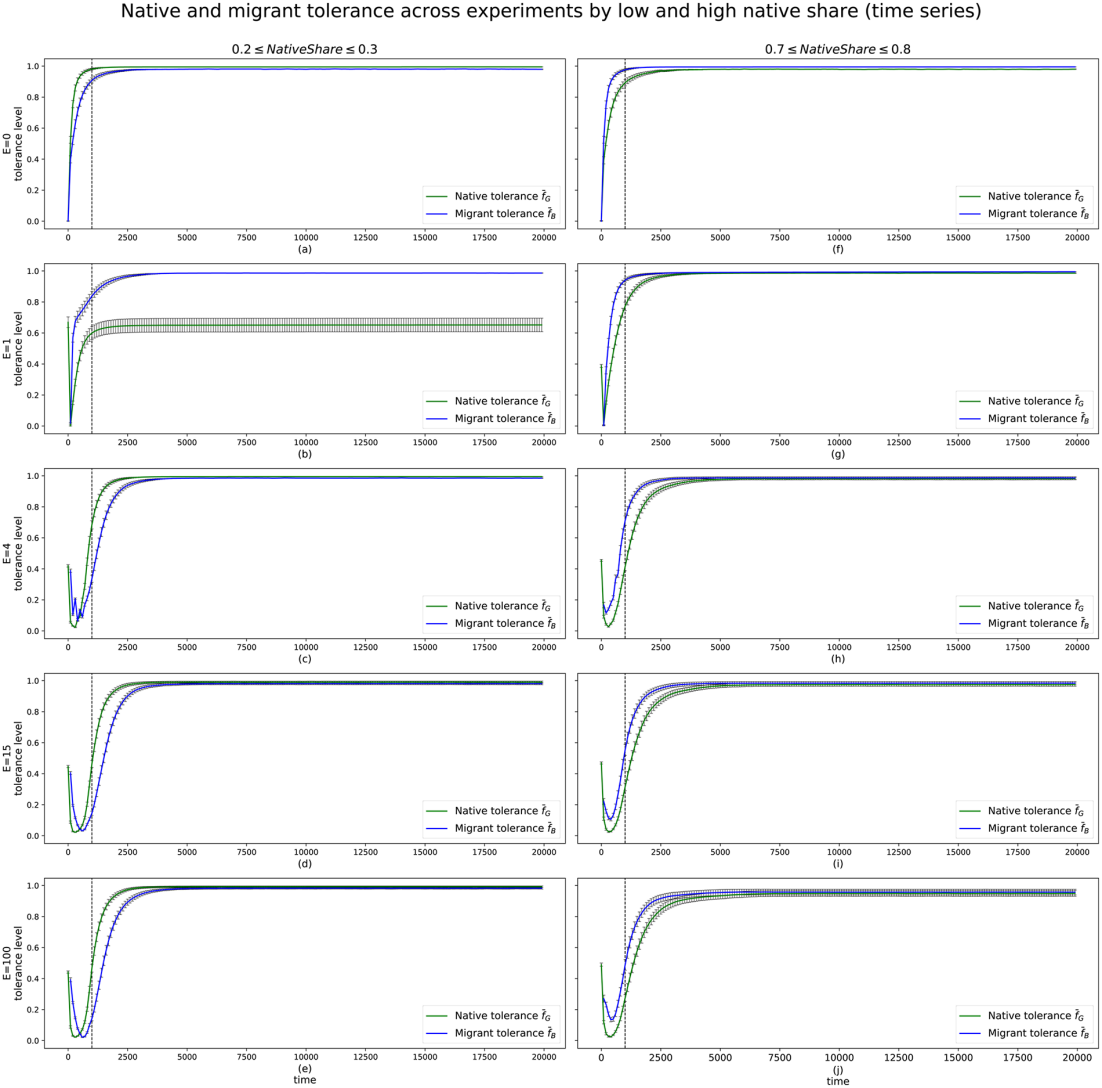
Time series of native tolerance ${\bar{f}}_{G}$ (green) and migrant tolerance ${\bar{f}}_{B}$ (blue), by treatment, filtered by extreme values of the native-share initial condition (0.2 ≤ *NatShare* ≥ 0.7). The error bars (black) show the 95% confidence interval for the mean of the $\bar{f}$ values across independent simulation runs and the range of Δ*f* that leads to convergence. The dashed line at *t*_*mig*_ marks the end of migration waves.

The tolerance of natives ${\bar{f}}_{G}$ (henceforth: ‘native tolerance’) and migrants ${\bar{f}}_{B}$ (henceforth: ‘migrant tolerance’) over time is plotted in Fig 4). An important variable affecting tolerance behaviour is *NatShare*, which determines the final size of the native share of the population. Fig 4 shows tolerance levels over time by the high and low values of native share, grouped into *NatShare* ≤ 0.3 and *NatShare* ≤ 0.7 respectively.

In the case of low native share, under no migration (*E* = 0) conditions, natives and migrants behave similarly (Fig 4a and 4f). The majority group is slightly less tolerant. The differences are visible at first, but nearly converge after *t* = 2500. The differences are very small, but still significant.

The case of *E* = 1 stands out from the rest. When *NatShare* is low (Fig 4b), natives never recover fully from the initial shock of migration. As with all other cases, native tolerance drops sharply and quickly recovers, but not exceeding ${\bar{f}}_{G}=0.7$ for the remaining time. The reverse scenario of high *NatShare* (Fig 4g) is visibly different: natives recover and reach near-total tolerance, along with migrants. These values are slightly higher than those observed in Fig 4f, with no migration. The reason that the *E* = 1 treatment is so different from the rest is that in the case of low *NatShare*, the proportion of natives to migrants is flipped immediately: native agents who constitute 20% of the final population make up 100% of the pre-migration population. Because of the large number of migrants coming in, many migrants are immediately exposed to a majority of migrants, presenting a shock not just to parts of the population, but to most of it. As no migrants had been present before, natives have not clustered into groups which could at least ‘shield’ the inner part from the effects of the one-off migration (this is because segregation behaviour is triggered by unhappiness rather than absence of out-group members, see section 2.1). Because natives suddenly find themselves in a minority and most of the agents become unhappy, they will move into areas with fewer migrants. The sudden influx has prevented even moderate natives to adapt to their changing neighbourhood, and as they start to segregate away, not enough tolerant natives remain to become happy and increase their tolerance levels. The sudden minority is an important element of this behaviour. As the high *NatShare* situation shows (Fig 4g), a one-off influx does not reduce long-term native tolerance when natives are in a majority even after the large migration wave. In contrast, natives in *E* = 0 treatments are exposed to migrants from the very beginning: they experience unhappiness and will segregate to improve it, creating pockets of happy and intolerant natives surrounded by tolerant natives that are exposed to migrants and shield the intolerant parts of their population group from exposure. This process is not achieved in *E* = 1 treatments, where previous absence of migrants has meant that native tolerance had started to decline.

At *E* = 4 (Fig 4c and 4h), the shocks from each migration wave is visible. When *NatShare* is low (Fig 4c), migrant tolerance is low when migration is still occurring. The increases in tolerance at each migration wave is due to the random initialisation of tolerance for new migrants. Effectively, each wave presents an opportunity to tip the tolerance balance within the population. This is not achieved until the fourth and last wave of migration has arrived at *t* = 700. Natives are visibly affected by the influx of migrants. During the first two migration waves, native tolerance ${\bar{f}}_{G}$ drops to near-zero, before increasing sharply to above 0.6 at *t* = 1000. Beyond this point, both natives and migrants transition to a majority tolerant society of ${\bar{f}}_{G}>0.98$ by *t* = 5000.

For *E* = 15 (Fig 4-d and 4-i) and *E* = 100 (Fig 4-e and 4-j) the overall pattern is very similar. The migration waves are no longer as visible on the graphs, as the size of waves is not large enough to upset the overall population. Both natives and migrants will first experience a drop to low levels of tolerance, and recover quickly as more waves arrive, reaching their peak. Higher numbers of migration waves increase the time required to reach convergence of peak tolerance when *NatShare* is high (Fig 4-i and 4-j). When *E* = 4, the peak is reached by *t* = 5000. When *E* = 100, this requires an additional 2,500 ticks. This means that for longer periods of time, the population groups are not as tolerant. The higher convergence times are also visible in Fig 3.

A notable difference between natives and migrants in the *E* = 15 and *E* = 100 cases is that natives will always drop their tolerance to near-zero, regardless of their population share. Migrants mirror this pattern only when they are in the minority (Fig 4-d and 4-e). When migrants are a majority (Fig 4-i and 4-j) their lowest tolerance is above 0.1. If natives and migrants behaved the same way, the graphs on the left should be mirrored by the graphs on the right. Lastly, when *E* = 100 and *NatShare* is high (Fig 4-j), the maximum tolerance levels are not as high compared to cases with fewer migration waves. The variance increases as the number of waves increases, suggesting a less settled pattern of tolerance.

### 3.4 Cross-sectional analysis

In this section we analyse the dependent variables listed in Table 5 across a total of 15,000 independent simulation runs, drawing parameters from the distributions in Table 1.

**Fig 5:**
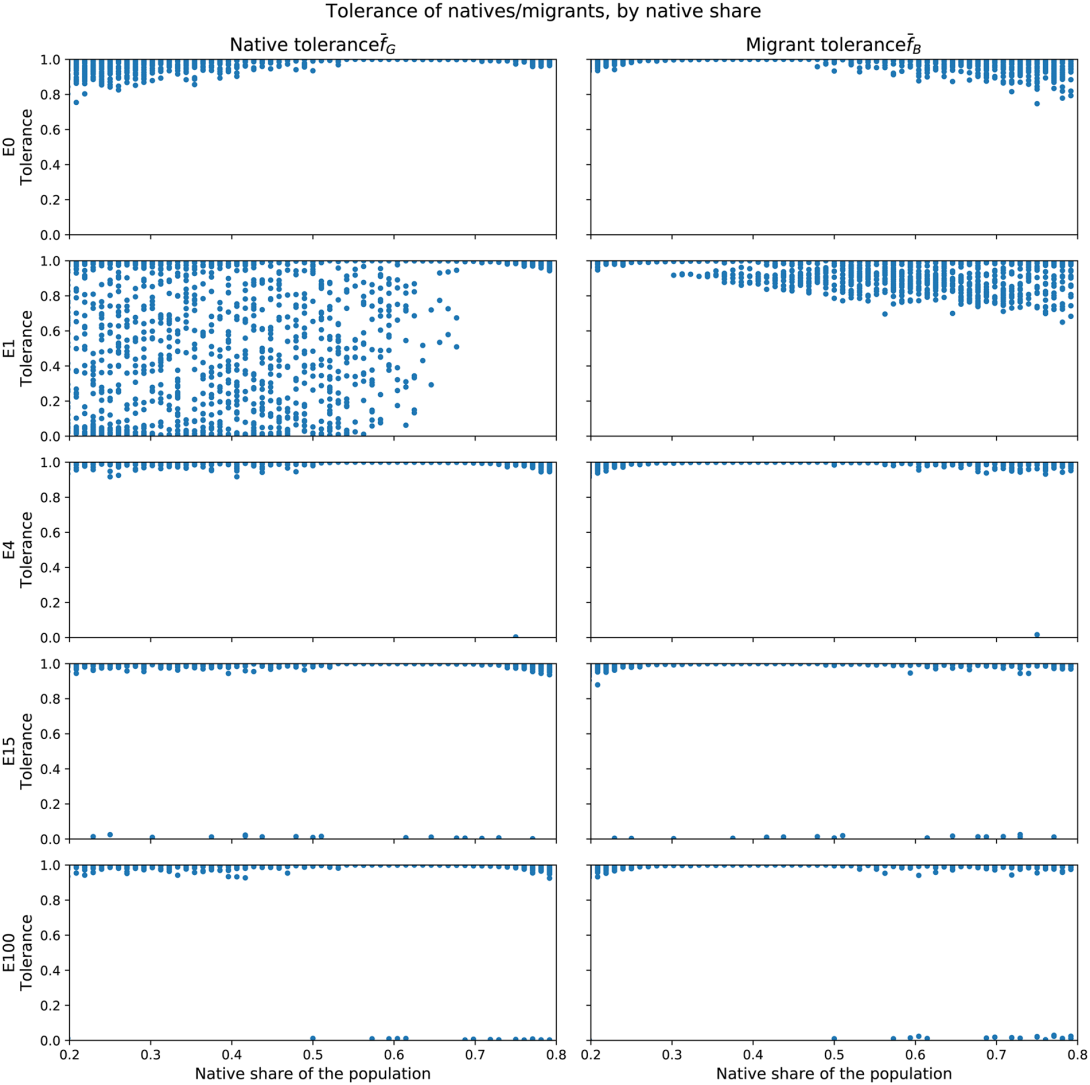
Scatter plots, by treatment, of native tolerance (${\bar{f}}_{G}$) and migrant tolerance (${\bar{f}}_{B}$) against native share of the population (*NatShare*), in steady-state at *t* = *t*_*max*_.

Fig 5 shows a scatter-plot of the average final tolerance of each group against the native-share initial condition (*NatShare*), subdivided by treatment. The first column shows the tolerance of natives ${\bar{f}}_{G}$ and the second column depicts the tolerance of migrants ${\bar{f}}_{B}$. Outcomes from the five immigration treatments from Table 4 are each shown on a separate row.

Because both natives and migrants share the same decision-rule for adapting their tolerance (algorithm 2), our initial intuition was that all the graphs would simply be mirrored. The control treatment with no migration, *E* = 0, shows this mirroring pattern for both native and migrant agents: when natives are in a minority, their tolerance is more varied, dropping to 0.8. The same behaviour is observed for migrants when they are in the minority.

When migration occurs only once (*E* = 1), natives and migrants differ markedly in their tolerance behaviour. Part of the pattern is still mirrored: natives that constitute the vast majority of agents (*NatShare* ≥ 0.7) are very tolerant, as are migrants when *NatShare* ≤ 0.3. When natives are in majorities smaller than 0.7, the native tolerance splits: a large number of cases see very high native tolerance, and very low tolerance. Medium levels of ${\bar{f}}_{G}$ are observed throughout. A part of this pattern is reflected in the earlier time-series of native tolerance in Fig 4. Migrants don’t diverge in their behaviour as much, although their tolerance starts to vary more as well, never dropping below 0.6. Migrants in minorities cope better than natives in minorities when *E* = 1.

This pattern does not apply for the cases *E* > 1. When *E* = 4, both natives and migrants are very tolerant with the exception of one outlier each, where tolerance is at or near zero. When *E* = 15, more cases of intolerant natives and migrants occur throughout the range of *NatShare*. When *E* = 100, those low-tolerance cases only appear at *NatShare* ≥ 0.5. Thus, when the number of migration waves is very high and natives form the majority of the population, more cases of intolerance occur. Tolerance levels always verge on the extreme ends of the scale.

The different types of migration flows modelled by the treatments in Table 4 do not significantly affect the broad functional relationship between tolerance ($\bar{f}$) and native-share (*NatShare*) if *E* > 1. The high variance of native tolerance at *E* = 1 is likely due to the fact that the one-off migration wave is so disruptive that for large parameter ranges, natives do not recover their tolerance.

High tolerance within very small groups is likely due to the fact that with so few agents, no coherent group can form, and thus all free-moving agents become more and more tolerant as they have no homogeneous neighbourhood to escape into. This line of reasoning is intuitive for migrants in general, since they arrive in smaller batches. The high variance of tolerant minority migrants could be down to their initial placement. Depending on where their clusters are located, they may find a cluster large enough to ensure lower tolerance levels. Those that roam the map on their own will exhibit high tolerance levels.

**Fig 6:**
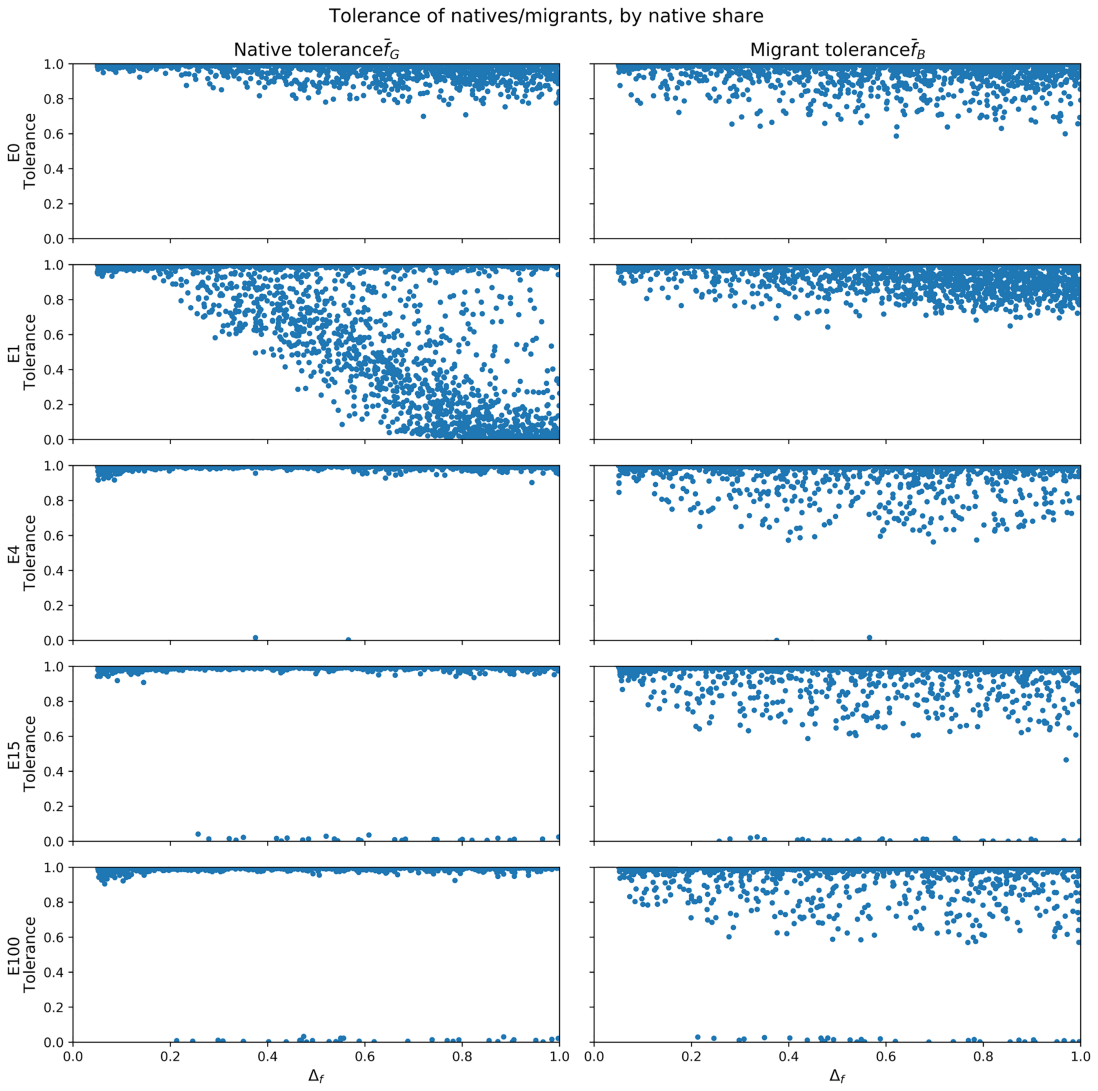
Scatter plots, by treatment, of native tolerance (${\bar{f}}_{G}$) and migrant tolerance (${\bar{f}}_{B}$) against the rate of change of tolerance (Δ*f*), in steady-state at *t* = *t*_*max*_.

As Δ*f* affects how quickly agents change their tolerance, we investigate its effects on agent tolerance as well. Fig 6 presents the same variables as Fig 5, this time with Δ*f* as the independent variable instead of *NatShare*. The overall patterns are very similar: migrant and native tolerance are very high in cases of *E* = 0, 4, 15 and 100, with an increasing number of low-tolerance agents as the number of migration waves increases. Again, *E* = 1 is a clear outlier. We recall that *NatShare* values up to 0.6 correlate with a high variance of native tolerance. The majority of agents are either extremely tolerant or intolerant, but in contrast to the other cases of *E*, many moderate natives exist, too. At *NatShare* levels above 0.7, tolerance once again dominates. Δ*f* as an independent variable affects ${\bar{f}}_{G}$ differently: as Δ*f* increases, native tolerance decreases. In other words, when natives can change their tolerance more quickly, many of them become more intolerant. However, just like *NatShare*, a proportion of natives stay very tolerance throughout all values of Δ*f*. As Δ*f* increases past 0.6, the variance of tolerance levels increases. More agents assume moderate tolerance levels- however, the majority reside on either side of the extreme. As discussed above, *E* = 1 deviates from the rest of the findings, and only does so for native agents. Fig 6 shows that Δ*f* is very important in determining the overall tolerance levels of natives in the case of *E* = 1: when agents are very slow to change their minds (Δ*f* ≤ 0.2), they change too slowly in order to express the experience of the large migration wave as low tolerance. When natives can react quicker (that is, they need fewer rounds to reach a new level of required in-group neighbours), they start to decrease their tolerance levels. It should be noted that until Δ*f* of 0.5, overall tolerant natives are still in the majority and that no natives below a ${\bar{f}}_{G}=0.5$ exist. For the other cases of *E* however, Δ*f* does not show such an impact.

### 3.5 Bimodality analysis

**Fig 7:**
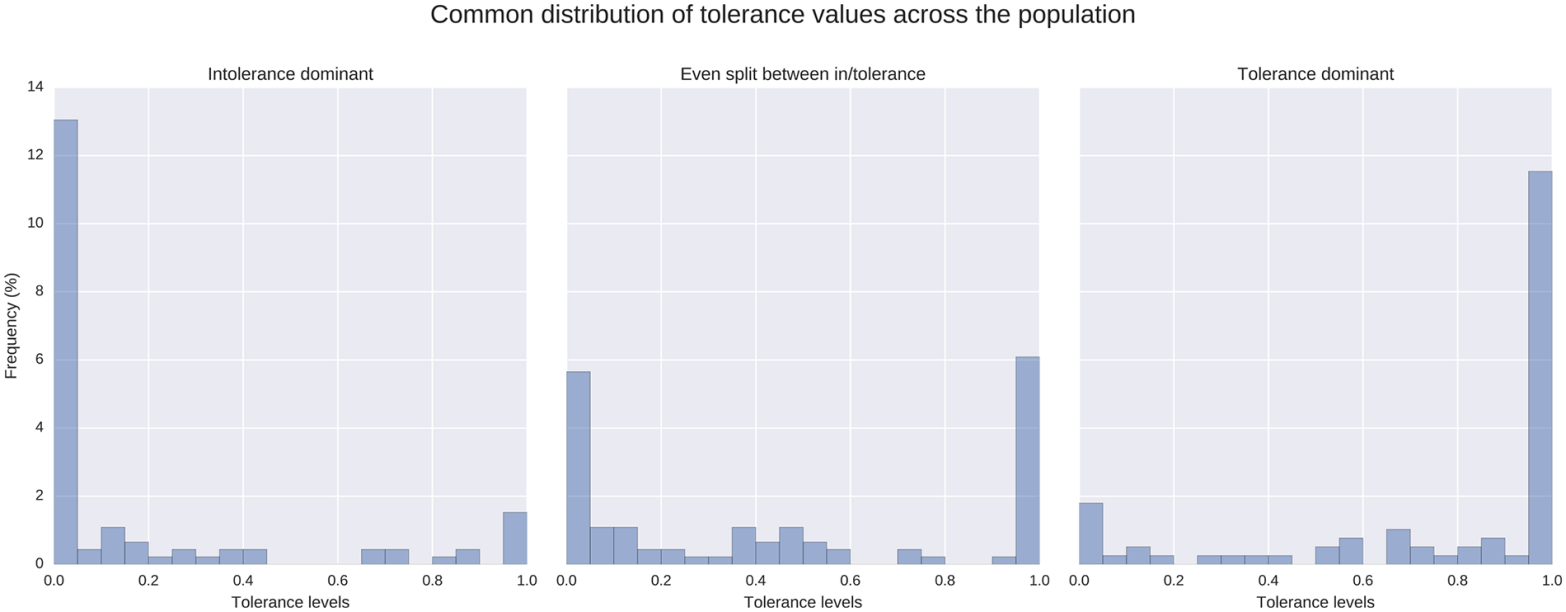
Histograms of the three most common distributions of final tolerance levels *f*_*i*_. Intermediate tolerance is infrequent when tolerance levels are polarised.

Both the cross-sectional and the time-series analysis indicate highly polarised tolerance levels. Fig 7 shows a histogram of tolerance values *f*_*i*_ across the population at the end of representative simulation runs. In these cases, tolerance values are concentrated at both extremes of the distribution, and the distribution is bimodal. Depending on the parameters, the split can vary between 30-70 and 70-30, with less than 10% of agents taking more moderate tolerance values. Thus parameters can determine the extent to which a population leans to the very tolerant or very intolerant, but the overall pattern remains that of a deeply divided society.

**Fig 8:**
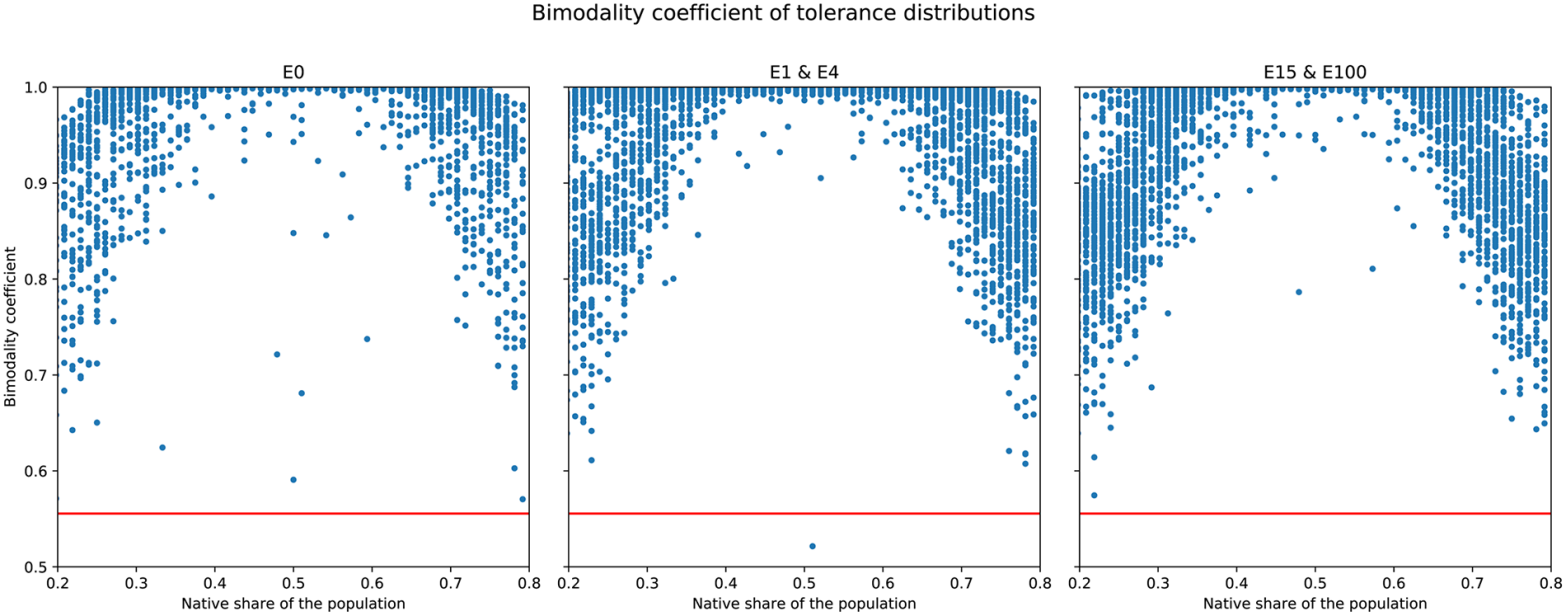
Scatterplot of the bimodality coefficient of the tolerance distribution *β*_*f*_ at the end of simulations, against native share of the population (*NatShare*). Treatments were merged here due to their similarity. The critical value $\beta_{f}>\frac{5}{9}$ is denoted by the horizontal red line.

Fig 8 shows a scatterplot of the bimodality coefficient of tolerance *B*_*f*_ against the native share of the population across all simulation runs. Regardless of the experimental setup, the bimodality coefficient is almost always above the critical value $B_{f}>\frac{5}{9}$, denoted by the red horizontal line. Due to their similarity, *E* = 1 and *E* = 4, as well as *E* = 15 and *E* = 100 were grouped together. Bimodality is lowest when native share is either $<\frac{1}{3}$ or $>\frac{2}{3}$ of the population. In cases with migration, bimodality drops earlier compared to the control. In a large number of cases, bimodality is nearly at 1 for the mid-range values of native share, illustrating its strong polarising effect on the population. The sharp drops in bimodality near the critical value are caused mainly by very low values of Δ_*f*_.

**Fig 9:**
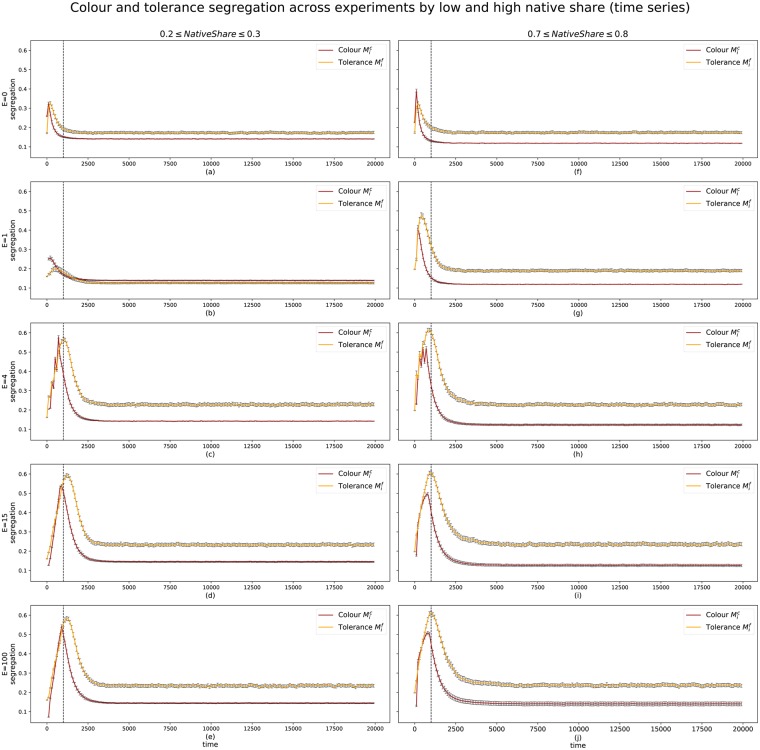
Time series, by treatment, of the segregation levels of colour $M_{t}^{c}$ (black), and tolerance $M_{t}^{f}$ (yellow), filtered by extreme values of the native-share initial condition (0.3 ≤ *NatShare* ≥ 0.7). The error bars show the 95% confidence intervals of the mean of *M*_*t*_ across simulation runs. The dashed line at *t*_*mig*_ marks the end of migration waves.

A divided society of agents is typical for a Schelling model, since agents are intrinsically homophilic by construction. However, divisions in a Schelling model are based on colour, or in this case, native or migrant status. The segregation of *tolerance* in this case is higher: Fig 9 shows the Moran’s I of spatial autocorrelation for both colour-based segregation *M*^*c*^ (as typically measured in a Schelling model) and for tolerance-based segregation *M*^*f*^. Again, the results are grouped into both ends of native share values and broken down by immigration treatment. The long-term segregation levels for out-groups (colour) are consistent throughout all cases, including the control (Fig 9-a and 9-f), never reaching *M*^*c*^ = 0.2. The values are not much higher than the values observed when movement is random (i.e. 0.1 ≤ *M*^*c*^ ≤ 0.15). Schelling models have a baseline *M*^*c*^ value because some segregation exists by pure chance of agents’ position at any point in time, suggesting that adaptive agents can circumvent segregation of colour to a large extent.

By contrast, segregation of tolerance attitudes, *M*^*f*^ is higher at the end of each simulation for most treatments of *E* and levels of *NatShare*. The exception is *E* = 1 and low *NatShare* (Fig 9-b), which sees both colour and tolerance segregation at similar low levels.

The differences between *M*^*c*^ and *M*^*f*^ are more pronounced when *NatShare* is high; (Fig 9-h, 9-i and 9-j) need slightly longer than cases with low *NatShare* (Fig 9-c, 9-d and 9-e). In the case of *E* = 4 (Fig 9-h), each migration wave is a visible shock to the existing tolerance level. Each drives *M*^*f*^ and *M*^*c*^ up. The same pattern is true of *E* > 4 (Fig 9-i and 9-j), but the smaller waves leave less distinct marks. As seen previously with tolerance developments in Fig 4, the values change most during the arrival of migration waves, and settle once the final wave has arrived. Convergence of segregation values is reached before *t* = 5000, where cases of *E* > 1 and high *NatShare* (Fig 9-h, 9-i and 9-j) need slightly longer than cases with low *NatShare* (Fig 9-c, 9-d and 9-e). Long-term levels of tolerance segregation are at or above 0.2 when *E* > 1, whereas no migration shows lower levels of *M*^*f*^.

The higher level of attitude-based segregation is interesting because agents do not actively seek out tolerant or intolerant neighbours; in fact, they are oblivious to the tolerance attitudes of their neighbours. The decision rule described in Algorithm 2 drives adjustment of attitudes, but not an open choice of out-group neighbourhood, as is the case with agent colour. The connection between diverse neighbourhoods and adjustments of tolerance seems to cause an unintended, much larger segregation than that of out-groups. Previous work including segregation of preferences have found the same effect (see [27] and [19]), but the scale of *M*^*f*^ is much lower in this adaptive model (see [19] for more insight into *M*^*f*^ in a non-adaptive model).

**Fig 10:**
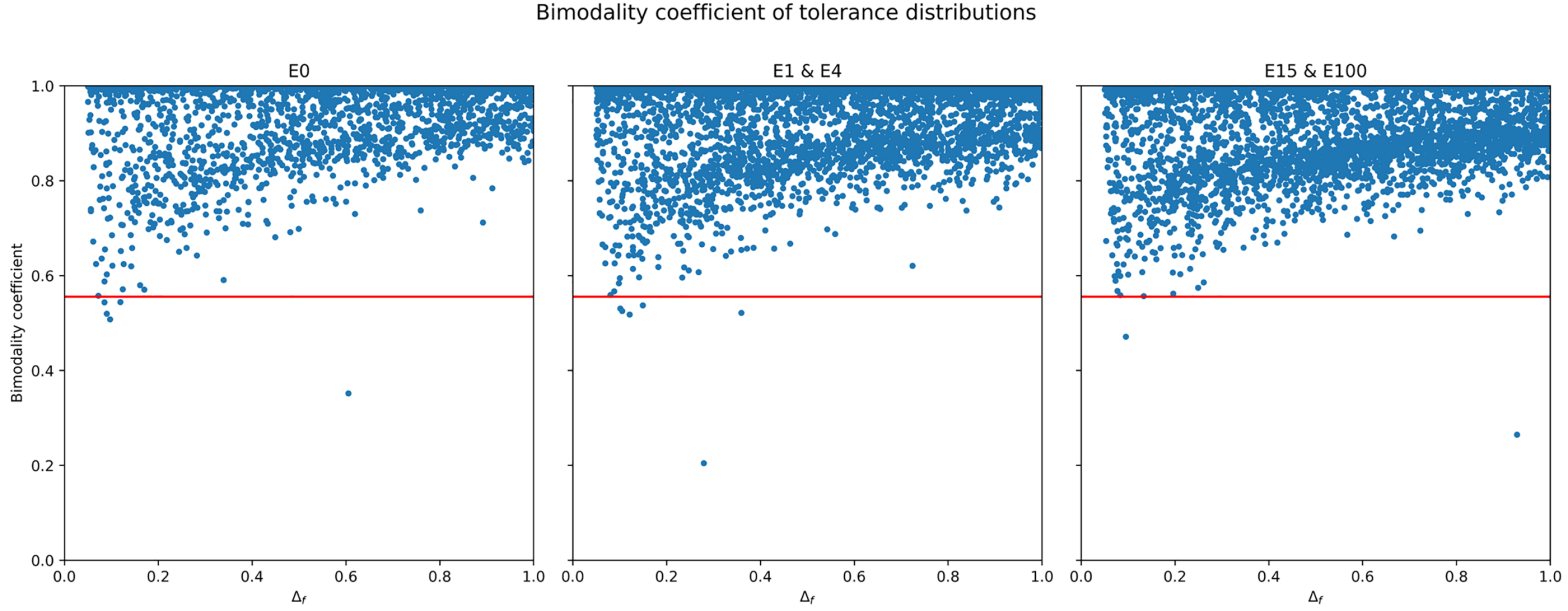
Scatterplot of the bimodality coefficient of the tolerance distribution *β*_*f*_ at the end of simulations, against the rate of change of tolerance (Δ_*f*_). Again, treatments were merged due to their similarity. The critical value $\beta_{f}>\frac{5}{9}$ is denoted by the horizontal red line.

Fig 10 shows the bimodality coefficient of tolerance just like Fig 8, this time against Δ_*f*_, the rate of change of tolerance. As in the case of *NatShare*, the vast majority of cases reside above the critical value of $\beta_{f}>\frac{5}{9}$, with only a handful of cases below the red line. The strong bimodality of tolerance throughout all cases of *E* is also upheld for the range of Δ_*f*_, showing that even at very low levels of Δ_*f*_, bimodality is still hight. Overall, lower values of Δ_*f*_ lead to lower levels of bimodality (yet above the critical value). That agents who can change their tolerance more quickly tend towards higher bimodality levels is intuitive: the ability to change tolerance quickly can push agents towards the extreme ends more easily. However, we recall as shown in Fig 6, Δ_*f*_ alone does not determine the split between high and low tolerance agents.

## 4 Discussion

Our modelling work contributes insights both to sociology and political science, which we discuss in turn below.

From the perspective of sociology, our results highlight the importance of minority and majority situations, which is corroborated by other studies [20]. Migration matters, but minority-majority population shares mediates the effects strongly, especially in the short-term. The differences are particularly pronounced for extreme values of the population share, and the effects can be both negative as well as positive. The importance of population share is in line with [8]’s suggestion that the respective standings of each out-group in society plays an important role in contact situations. Our model suggests that the mere size difference leads to logistic situations that affect inter-group contact. This result also resonates with some of the findings in the empirical literature. For example, [28] find that majority groups experience a greater decline in prejudice as the result of contact compared to minority groups. The status of groups influences the potential perceived threat [6]. Dominant groups might fear a loss of privilege, the subordinate groups might worry about oppression [6, p.195].

From the perspective of political science, we have shown that simple yet theoretically-plausible behavioural rules can give rise to a polarisation in tolerance towards out-groups, and self-assortment of the population into tolerant and intolerant clusters. Neither behaviour is built into the model from the outset, but rather these are emergent behaviours that arise from a subtle interaction between adaptive tolerance and movement of the population. Crucially, divisions of native-migrant groups do not necessarily predict divisions in tolerance. Rather, intermediate tolerance levels are inherently unstable when movement and tolerance-adaptation interact. Sometimes individual agents transition from one extreme to the other, but most agents remain either very tolerant or intolerant.

This assortment of the population into communities of similar tolerance is highly reminiscent of the current political landscape in the UK, the US and the many European countries that have experienced a surge in populist parties. These new developments have shifted the divide between economic left and right to more sociocultural divisions. In Britain, those who voted to leave the EU in the 2016 referendum were characterised by social conservatism, nationalism and low levels of political trust, whereas remain voters were more likely social liberals, cosmopolitan and high on trust values [29]. Similar divisions are visible for Trump and Clinton voters in the US. Social and economic ideologies change how voters perceive social and economic issues [30]. Non-economic issues have become increasingly important for political parties in the West [31], and populist parties and candidates appeal on the basis of fears about immigration, sovereignty, and security. Both liberals and conservatives are subject to increased prejudice towards the respective political out-group [30], but the rise in populist narrative builds on immigrant narratives especially in the Netherlands and in the Brexit referendum in Britain [29]. The Leave majority was highest in areas that were the least diverse or featured high numbers of working-class voters; but also in areas which had experienced rapid demographic change as the result of immigration in the past ten years [29].

Moreover, the *mechanism* that causes self-assortment along the tolerance axis also has a plausible real-world analogue. Within the model, polarisation of tolerance is caused by the shield-and-buffer dynamic described in section 3.1 which prevents clusters of intolerant agents from one group from being exposed to out-group agents. This shield-and-buffer dynamic might offer one potential explanation for the political bimodality found today. Cosmopolitan areas of a country are generally populated by people that are more tolerant of migrants; and migrants that reside in these areas are generally tolerant in turn [5]. However, if the migrant diaspora exceeds a certain size, it could potentially sustain a sub-culture that is not dependent on integration with the host population. If non-migrants that live outside these areas have no direct contact with migrants, or the migrant fraction of the population is too small to provide sufficient contact situations, and peoples’ out-group tolerance increases or decreases as described by contact and threat-theory, then polarisation would ensue, just as it does in our model.
